# Metabolomic signatures of early pathway disruptions and clinical outcomes in community-acquired pneumonia: a prospective case-control study

**DOI:** 10.1186/s12890-026-04503-9

**Published:** 2026-07-23

**Authors:** Souvik Chaudhuri, Varashree Bolar Suryakanth, Shruthi Rao, Ashritha A Udupa, Monalisa Biswas, YS Phaneedra Mallimoggala, Thejesh Srinivas, Bharatkumar Appasaheb Patil, Ranajit Das, Nitin Gupta

**Affiliations:** 1 Department of Critical Care Medicine, Kasturba Medical College, Manipal Academy of Higher Education, Manipal, India; 2 Department of Biochemistry, Kasturba Medical College, Manipal Academy of Higher Education, Manipal, India; 3 Division of Data Analytics, Bioinformatics and Structural Biology, Yenepoya Research Centre, Yenepoya (Deemed to be University), Mangalore, Karnataka India; 4 Department of Infectious Diseases, Kasturba Medical College, Manipal Academy of Higher Education, Manipal, India

**Keywords:** Community-acquired pneumonia, Severe CAP, Untargeted metabolomics, Metabolic pathway dysregulation, Mechanical ventilation

## Abstract

**Background:**

The disruption of metabolic pathways in patients with community-acquired pneumonia (CAP) may provide insights into disease progression. We aim to identify early disruption in the serum metabolome of CAP patients using untargeted metabolic approaches.

**Methods:**

We conducted a single-centre, prospective case-control study involving 105 participants (70 with CAP and 35 healthy controls), between January 2025 and November 2025. We also performed a subgroup exploratory metabolomic analysis among the 70 CAP patients, comparing 60 patients with severe CAP requiring ICU admission with the 10 non-severe CAP patients who did not require ICU admission. Untargeted serum metabolomics was performed using gas chromatography-mass spectrometry (GC-MS) to investigate metabolic dysregulation in patients with CAP. The analysis of the clinical data was performed using SPSS version 25.0. The data of metabolomics was analyzed using MetaboAnalyst version 6.0. After the preprocessing, log_10_ transformation, normalization and scaling, 93 GC-MS metabolites were retained from the initial 300 metabolites. Principal component analysis was done for unsupervised clustering, which was then followed by OPLS-DA for supervised group discrimination. Model performance was assessed using R²Y and cross-validation-derived Q² values, and model robustness and overfitting exclusion was further evaluated using permutation testing. Discriminatory metabolites were identified using VIP scores, fold change, log₂ fold change and univariate testing with false discovery rate correction where applicable. ROC analysis, heatmap-based clustering and pathway enrichment analysis were performed.

**Results:**

The variable importance in projection (VIP) scores showed that out of 93 untargeted metabolites studied using GC-MS, the greatest differences between the CAP patients and healthy controls were about malic acid (VIP:3.374, fold change 2.591), 4-hydroxybenzeneacetic acid (VIP:2.459, fold change 27.97), 2-hydroxybutyric acid (VIP:2.059, fold change 4.779), and 4-hydroxyphenyllactic acid (VIP:1.882, fold change 20.67). Key disrupted pathways in CAP patients included propanoate, glyoxylate, and dicarboxylate; tricarboxylic acid cycle; nitrogen; tyrosine, cysteine, and methionine; and amino acid pathways. Comparison between the severe CAP patients (ICU admission required) and non-severe CAP patients (no-ICU admission), VIP analysis identified boric acid, ethylene glycol, ethanimidic acid, linoleic acid, oleic acid, cholesterol, 3-hydroxybutyrate, and α-tocopherol as the principal discriminating metabolites, while pathway analysis revealed perturbations in glutathione metabolism, amino acid metabolism, the tricarboxylic acid cycle, pyruvate metabolism, and arachidonic acid metabolism.

**Conclusions:**

CAP patients demonstrated early disruptions of key metabolic pathways compared with healthy controls, spanning energy metabolism, amino acid pathways, redox balance, lipid inflammatory signalling, and gut–host metabolic axes. These findings highlight the potential utility of metabolomic profiling as an adjunctive clinical tool for early risk stratification and biochemical phenotyping in CAP.

**Trial registration:**

Clinical trials Registry of India (CTRI 2024/12/078631) registration was done 27.12.2024.

**Supplementary Information:**

The online version contains supplementary material available at https://doi.org/10.1186/s12890-026-04503-9.

## Background

Community-acquired pneumonia (CAP) is the most common cause of lower respiratory tract infection, with signifant hospitalizations worldwide [1]. It is the second most common cause of mortality in low- and middle-income countries [2] Research on clinical profiles, biomarkers, and metabolomics is imperative for precision therapy in the future [1]. Metabolomics allows an early window into the dysregulated biological pathways in diseases [3]. Recent reviews emphasize that metabolomics enables systematic identification of metabolic dysregulation in disease states, providing clinical insights with translational therapeutic relevance [4]. Metabolomics reveal dynamic changes in metabolic pathways well before the traditional biomarkers and clinical organ dysfunction become apparent to the clinician, and thus offer an early window for risk stratification [5]. The role of metabolomics for not only diagnosis, but also prognosis and response to therapy has been shown to hold promise in cardiovascular, cancer, neurodegenerative, and metabolic diseases [6]. Metabolomic profiling identifies metabolites predictive of disease progression, response to therapies, and clinical outcomes in respiratory disorders such as pneumonia and acute respiratory distress syndrome (ARDS) [7, 8]. Recent evidence suggests that pulmonary dysfunction is associated with amino acid dysregulation, which correlates with disease severity and metabolic stress [9]. Dysregulated amino acid metabolism predicts poorer outcomes in critically ill patients, and this is compounded by the fact that about 42% critically ill patients are at risk of malnutrition. A study on metabolomics involving amino acid metabolism, along with other untargeted metabolomics in CAP, may be an important topic of research [10, 11]. This intersection of metabolic alterations and nutritional risk underscores the need for a comprehensive, untargeted metabolic approach to define metabolic dysregulation in CAP patients.

CAP may lead to severity in disease progression, and even ARDS. There have been previous studies on the utility of metabolomics for prognostication of CAP outcomes in H1N1 CAP and bacterial CAP [12, 13]. However, the studies have focused on viral pneumonia, particularly H1N1 influenza pneumonia, with bacterial CAP used mainly as a comparator group rather than as the primary disease phenotype of interest. Other study in bacterial CAP have primarily used targeted lipidomics and identified lipid-based prognostic signatures, including alterations in lysophosphatidylcholines, phosphatidylcholines and acylcarnitines [12, 13].

There remains a limited insight into the early metabolic dysregulation that takes place in CAP patients in mitochondrial energy metabolism, amino-acid utilisation, redox balance, lipid-inflammatory signalling and host–microbiome interactions. Also, the spectrum of metabolic alterations in severe CAP patients as compared to non-severe CAP patients, irrespective of aetiology may aid in the identification of early pathway disruptions that are not captured by routine clinical biomarkers or CAP severity scores.

Untargeted metabolomics is in a unique position to address this gap because it provides a broad assessment of metabolites that reflect the downstream effects of infection, inflammation, tissue hypoxia, oxidative stress and host metabolic alteration. Unlike targeted approaches that quantify preselected metabolites or pathways, untargeted GC-MS metabolomics can detect a broader spectrum of biochemical alterations and generate new knowledge regarding disease mechanisms and severity-associated metabolic phenotypes. We aimed to identify early untargeted metabolomic alterations in patients with CAP as compared to healthy controls. The objective was to identify metabolite pathways and specific metabolites that are dysregulated early in illness in hospitalized CAP patients (within 24 h) compared with healthy controls, using an untargeted metabolomics approach. We also performed subgroup exploratory metabolomic analysis comparing the dysregulated metabolic pathways in the severe CAP patients (requiring ICU admission) as compared to the non-severe CAP patients (no ICU admission).

## Methods

This manuscript has been written in accordance with the Strengthening the Reporting of Observational Studies in Epidemiology (STROBE) guidelines.

### Study design and study setting

This study was a single-centre, prospective case-control study. CAP patients were recruited between January 2025 and November 2025. The ethical committee clearance (IEC 406/2024) was obtained before the commencement of the study. The patients were recruited after written informed consent was obtained. Written informed consent was also obtained from the healthy controls.

### Inclusion and exclusion criteria

We included adult patients aged 18 years or older. The diagnosis of CAP was based on the findings of a newly recognized pulmonary infiltrate(s) on imaging of the chest and at least one respiratory symptom (new or increased severity of cough, dyspnoea, new or increased production of sputum, chest pain which is pleuritic in nature) and at least one sign (fever ≥ 100.4 º F, hypoxia with oxygen saturation < 90%, lung sounds being abnormal like rales or rhonchi and leukocytosis) [14].

We excluded patients hospitalised for more than 48 h, who could be at risk of hospital-acquired pneumonia, patients with pulmonary tumours, interstitial lung disease, and pulmonary embolism.

The healthy controls were individuals aged 18–45 years who had visited the hospital for a routine health check-up. We excluded individuals with any comorbid illnesses, on any medications in the last 14 days, with any symptoms such as fever, cough, expectoration, or pain, or with signs such as rashes suggestive of acute illness.

### Definition of cases and controls

Cases were defined as adult patients with CAP aged > 18 years, as per the diagnostic criteria [14]. Controls were defined as adult individuals aged 18–45 years without any comorbidities, medication intake in the last 14 days, and without acute illness.

### Sample size

Sample size was calculated based on detecting a moderate effect size (Cohen’s d = 0.65) in metabolite levels between CAP patients and healthy controls using a two-sided independent samples comparison, with 80% power and α = 0.05 [15]. Using the formula for unequal group sizes with a 2:1 case–control ratio, the minimum required sample size was estimated to be approximately 30 controls and 60 cases. Considering the high dimensionality of untargeted metabolomics data and biological variability, an inflation factor of approximately 20% was applied to improve the model stability. Thus, the final sample size was set at 70 CAP patients and 35 healthy controls (total *n* = 105).

### Data collection

Seventy patients with CAP were recruited consecutively on the day of hospital admission, and the healthy controls were recruited during the same time period. The demographic parameters, co-morbidities, the scores for severity of CAP like pneumonia severity index score (PSI score), confusion, urea, respiratory rate, blood pressure and age ≥ 65 years (CURB-65) score, SMART-COP score (Systolic blood pressure, Multilobar infiltrates, Albumin level, Respiratory rate, Tachycardia, Confusion, Oxygenation, and arterial pH) were recorded. The PSI score is used to prognosticate outcomes CAP patients and determine low risk patients who are suitable for out-patient care. SMART-COP is used to identify patients at risk of requiring intensive respiratory or vasopressor support.

The sequential organ failure assessment (SOFA score), arterial blood gas, lung ultrasound score, Brixia chest x-ray score, and inflammatory biomarkers, C-reactive protein (CRP) and procalcitonin, were recorded at baseline. We performed comparative metabolomics analyses of CAP patients versus healthy controls using gas chromatography mass spectrometry (GC-MS).

### Sample collection and metabolomics analysis

#### Sample collection

The collection of blood samples from the CAP patients for metabolomic analysis was always within 24 h of hospital admission. Pragmatically, the samples were collected as early as feasible after diagnosis and enrolment, when the baseline investigations were also being sent. Sampling was not delayed ensuring fasting conditions, as early collection was prioritized to capture the initial metabolic state at hospital presentation and to avoid interference with urgent clinical management. Therefore, as this was a study involving hospitalized CAP patients, many of whom requiring acute care and presenting with different severity of illness, fasting status and time of day of sample collection could not be standardized. Healthy controls were recruited during routine health check-up visits during the same study period. After random blood sample collection, serum samples were processed according to the study protocol and stored at − 80 °C until GC-MS analysis.

#### Sample preparation

Frozen serum samples stored at -80 °C were thawed at room temperature. 300 µl of the sample were added to the centrifuge tubes, followed by 40 µl of the internal standard (Margaric acid) in each tube. Subsequently, 500 µl of 5% hydroxylamine hydrochloride and 400 µl of 2.5 M sodium hydroxide were added. The mixture was vortexed and incubated at room temperature for one hour. After incubation, 350 µl of 6 M hydrochloric acid (HCl) were added to each tube. Extraction was performed by adding 3mL of ethyl acetate, followed by centrifugation at 4400 rpm for 7 min. 1 mL of the obtained organic layer was then transferred to a new glass tube and evaporated to dryness under the stream of nitrogen gas at 60 degrees Celsius. For derivatization, the dried residue was treated with 100 µl of BSTFA (N, O-bis(trimethylsilyl) trifluoroacetamide) and 10 µl of TMCS (Trimethylchlorosilane), incubated at 80 °C for 40 min in a dry bath, then treated with 100 µl of ethyl acetate and vortexed. The derivatized samples were then transferred to autosampler vials and analysed by gas chromatography-mass spectrometry (GC-MS).

#### Instrument settings

One µl of the derivatized sample was injected into the gas chromatography (GC) coupled with Shimadzu Mass Spectrometer (MS) (GC/MS—QP2020 NX SHIMADZU, Shimadzu Corp., Tokyo, Japan). The GC equipped with a capillary column, SH-I-5Sil MS Capillary (30 m x 0.25 mm x 0.25 μm), was used for chromatographic analysis. The injector temperature was set to 280 °C (split mode, split ratio 30), with a column flow of 1.20 mL/min (Helium) and a total flow of 40.2 mL/min. The oven temperature programme was set to an initial temperature of 50 °C, held for 2 min, followed by an increase to 300 °C at 15 °C/min, with a hold time of 5 min and a total run time of 23 min. The ion source temperature was set to 230 °C, the interface temperature to 280 °C, and the solvent cut time to 3 min. Data was acquired in scan mode (35 to 550 m/z with a scan speed of 2000). The NIST library annotated identified metabolites.

#### Metabolite inclusion criteria and quality control

Peak metabolite detection and integration were carried out in the GC-MS processing software, applying a slope threshold of 100 and a peak width of 2. Compound identification relied on spectral matching against the NIST library (with allowed variation in ratio of reference ions set at/ below 30% as per standard guidelines), and features lacking a detectable peak or showing reference ion ratios outside the expected range were removed from the dataset. We retained only those metabolites present in at least 80% of samples, and where a given metabolite appeared as multiple features, the one with the highest peak intensity was kept while the rest were discarded to prevent duplication. This left us with 93 metabolites out of 300 metabolites per sample.

To ensure data quality, blank samples were run at the start and end of each analytical batch, allowing us to track contamination and carryover effects. Reproducibility was further assessed by running a randomly chosen subset of samples in triplicate throughout the analytical sequence.

During preprocessing, any metabolite missing in more than half of the samples was dropped from further consideration. For the remaining metabolites, missing values were filled in using the mean of that metabolite’s measured values prior to downstream statistical analysis.

#### Data pre-processing

The preprocessing of the metabolomics data was done before the PCA and OPLS-DA analysis. We excluded metabolites with > 50% missing values across the samples and excluded compounds obtained in column blank and solvent blank samples. Mean value imputation for each corresponding metabolite was performed for the remaining values that were missing (as per software settings in Metaboanalyst). The low-abundance features that had very low signal intensity were filtered based on the values of mean intensity, and the low variance features were excluded. We used the IQR method for excluding the low variance features. The data was then log-10 transformed to decrease the unequal variance and improve the distribution characteristics also. We did not perform any sample-wise normalization or scaling post log-transformation, as all the samples were processed and then analysed in a single batch with the same sample-preparation protocol, extraction volume, internal standards and GC-MS settings. Then, PCA was done as an unsupervised method for the evaluation of clustering patterns and the outliers. The supervised method was carried out by OPLS-DA in order to ensure maximum group separation and specifically identify metabolites that are contributing to the class discrimination. Since all the samples were analysed within the same analytical batch, the same GC-MS platform and the same sample-processing protocol application, we did not apply inter-batch correction. Signal-drift correction was not conducted because the analysis was not performed in multiple analytical batches. We applied the same preprocessing method uniformly to the CAP and healthy control samples.

### Statistical analysis

Statistical analysis of clinical variables was performed using IBM SPSS version 25.0 (IBM Corp., Armonk, NY, USA). The normality of the distribution of variables was tested using the Kolmogorov-Smirnov test. The mean and standard deviation (SD) or median and interquartile range (IQR) were reported.

For metabolomics analysis, MetaboAnalyst version 6.0 was used [16]. Metabolomic data were analyzed using a standardized untargeted workflow [17]. Data preprocessing included normalization and log_10_ transformation of metabolite intensities to reduce technical variability, account for the large dynamic range of metabolomics features and improve suitability for downstream univariate and multivariate statistical analyses [17]. A total of 93 metabolites spanning multiple metabolic pathways, including the tricarboxylic acid cycle, fatty acid metabolism, glucose metabolism, propanoate metabolism, glyoxylate and dicarboxylate metabolism, amino acid metabolism, ketone body metabolism, and related pathways, were included in the analysis. Univariate statistical comparisons between the CAP patients and healthy controls were performed to identify differentially abundant metabolites. Effect sizes were expressed as fold change (FC), and log₂ fold change (log₂ FC), and statistical significance was assessed using appropriate parametric or non-parametric tests, with adjustment for multiple comparisons where applicable.

Principal component analysis (PCA) was initially applied as an unsupervised method to assess data structure, identify clustering patterns, and detect potential outliers. Supervised multivariate modelling was subsequently performed using orthogonal partial least squares discriminant analysis (OPLS-DA) to maximize group separation and identify metabolites contributing to class discrimination. Model robustness was evaluated using cross-validation and permutation testing. Multivariate analyses were conducted to evaluate global metabolic differences between groups. Variable importance in projection (VIP) scores derived from OPLS-DA were used to rank metabolites according to their contribution to group separation, with higher VIP values indicating greater discriminatory power. VIP scores > 1 indicate that metabolites differ significantly between CAP patients and healthy controls. A fold change (FC) > 1 indicates that the metabolite level is higher in CAP patients, and an FC value of 2 implies that a particular metabolite is 2-fold higher in CAP patients than in controls. Scores plots were generated to visualize sample distribution and separation between CAP patients versus controls. Statistical analyses and visualizations were performed using MetaboAnalyst (version 6.0), and results were interpreted in the context of biologically relevant metabolic pathways.

To determine whether the metabolite differences observed between CAP patients and healthy controls were significant, after accounting for the age differences between the CAP patients and the healthy controls, an age-adjusted linear regression approach was performed for a selected set of discriminatory metabolites based on highest VIP values. The metabolites included in the age- adjusted regression analyses were selected from the CAP versus healthy control discriminatory metabolite panel identified in the primary metabolomics analysis. These metabolites represented the key dysregulated metabolites ranked by VIP score, log₂ fold change, and pathway relevance, and were therefore further evaluated to assess whether their association with CAP status persisted after adjustment for potential demographic confounders. Raw peak-area values of the metabolites were log_10_-transformed. A separate ordinary least-squares regression model was then built for each metabolite with age as an adjusting covariate. Beta coefficients, standard errors, and two-sided p-values were noted. Given the number of metabolites tested, multiple comparisons were addressed through Benjamini–Hochberg false discovery rate correction, with FDR-adjusted *p* < 0.05 was considered as significant. Pathway enrichment analysis was performed using the Pathway Analysis module of MetaboAnalyst version 6.0. The list of identified discriminatory metabolites was uploaded into the module. Metabolite names were checked and verified before analysis. The metabolites were then mapped to metabolic pathways using the Kyoto Encyclopedia of Genes and Genomes (KEGG) database. The Homo sapiens pathway library was selected.

Pathway enrichment analysis was used to identify pathways that were over-represented among the significant metabolites. Pathway topology analysis was also performed to assess the relative importance of the mapped metabolites within each pathway. The results were shown as pathway scatter plots generated by MetaboAnalyst. In these plots, the p value reflected pathway enrichment, while the pathway impact reflected the biological relevance of the pathway. Pathways with higher enrichment and higher pathway impact were considered more relevant and were interpreted in relation to CAP pathophysiology.

## Results

Seventy CAP patients and 35 healthy controls were included in this study. Table 1 shows the demographic characteristics and the clinical variables of the CAP patients. The median age of CAP patients was 70 (61.75-76) years. The mean Charlson Comorbidity Index (CCI) score was high at 5.40 ± 2.2, indicating a high comorbidity burden. Microbiological diagnosis of the respiratory samples confirmed the diagnosis in 6 of 70 patients (8.57%). Among these, *Haemophilus influenza* (*n* = 2/6, 33%) and *Klebsiella pneumoniae* (*n* = 2/6, 33%) were identified pathogens, along with *Streptococcus pneumoniae* (*n* = 1/6, 16.7%) and Influenza A H1N1 (*n* = 1/6, 16.7%). The CAP patients had a high severity of illness, as reflected by a PSI score of 125 (IQR 104.5–143.75) and a mean SMART-COP score of 4.44 ± 2.01 (Table 1). Respiratory failure was evident with a median PaO₂/FiO₂ ratio of 210 (IQR 152.75–290.50). A substantial proportion required advanced respiratory support, including non-invasive ventilation (51.4%) and invasive mechanical ventilation (40%), with 85.7% requiring ICU admission.

**Table 1:** The baseline variables in CAP patients and the demographics in healthy controls

Variables	Mean ± SD / Median (IQR) CAP and severe CAP patients (*n* = 70)	Healthy controls (*n* = 35)
Age	70 (61.75-76)	35.0 ± 8.77
Gender (male)	34 (48.6%)	13 (37.1%)
CCI	5.40 ± 2.21	
Smokers	19/65 (29.23%)	
SOFA score	4.5 (2–6)	
SMART-COP score	4.44 ± 2.01	
PSI score	125 (104.5-143.75)	
CURB-65 score	2 (1–2)	
Brixia CXR score	8 (6–10)	
LOS (hospital) days	11 (7.75-17)	
ICU admission required (%)	60 (85.7%)	
ARDS progression (%)	23 (32.9%)	
Mortality	15 (21.4%)	

*Legend* *CAP* community-acquired pneumonia, *CCI* Charlson Co-morbidity Index score, *SOFA* Sequential Organ Failure Assessment, *SMART-COP score* (Systolic blood pressure, Multilobar infiltrates, Albumin level, Respiratory rate, Tachycardia, Confusion, Oxygenation, and arterial pH), *PSI score* Pneumonia Severity Index Score, *CURB-65 score* confusion, urea, respiratory rate, blood pressure and age ≥ 65 years, *LOS* length of stay, *ARDS* Acute Respiratory Distress Syndrome; AKI: Acute Kidney Injury

**Fig. 1:**
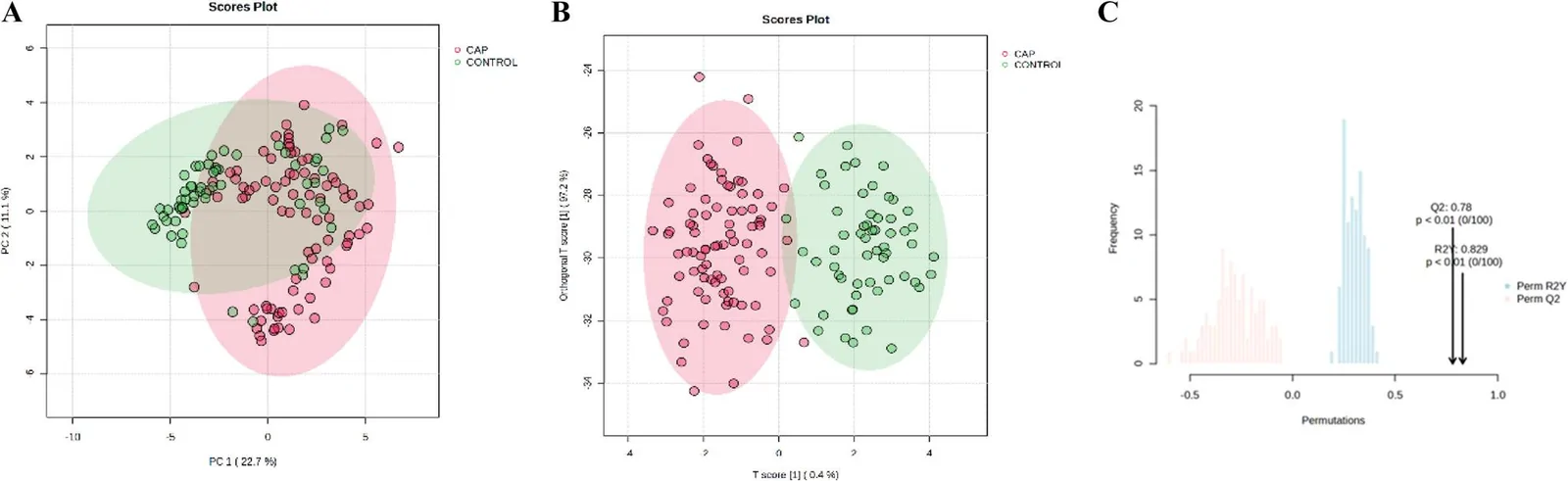
Analysis of metabolic profiles in community-acquired pneumonia (CAP). **A** Principal component analysis (PCA) score plot illustrating the global metabolic distribution of CAP patients and healthy controls. **B** Orthogonal partial least squares discriminant analysis (OPLS-DA) score plot demonstrating distinct metabolic separation between CAP patients and controls. **C** Permutation test validating the robustness and reliability of the OPLS-DA model. **A** PCA demonstrates overall metabolic distribution with partial group separation. **B** OPLS-DA shows clear discrimination between groups, indicating distinct metabolic profiles; the horizontal axis represents disease-related variation, and the vertical axis reflects within-group variability. Ellipses denote sample distribution. **C** Permutation test validating the OPLS-DA model. Higher R² and Q² values in the original model compared to permuted models (*p* < 0.01) confirm robustness and exclude overfitting. Legend-Score plots showing metabolic differences between CAP patients (red) and healthy controls (green), with each point representing an individual sample

We identified 93 metabolites in total during the untargeted GC-MS analysis. Unsupervised Principal Component Analysis (PCA) initially assessed the data structure, followed by OPLS-DA, which demonstrated distinct separation between CAP patients and healthy controls. The OPLS-DA model’s validity was confirmed by a permutation test (100-fold), giving a Q2 of 0.78, indicating greater predictive relevance and minimal overfitting. (Fig. 1, Panels A-C).

**Fig. 2:**
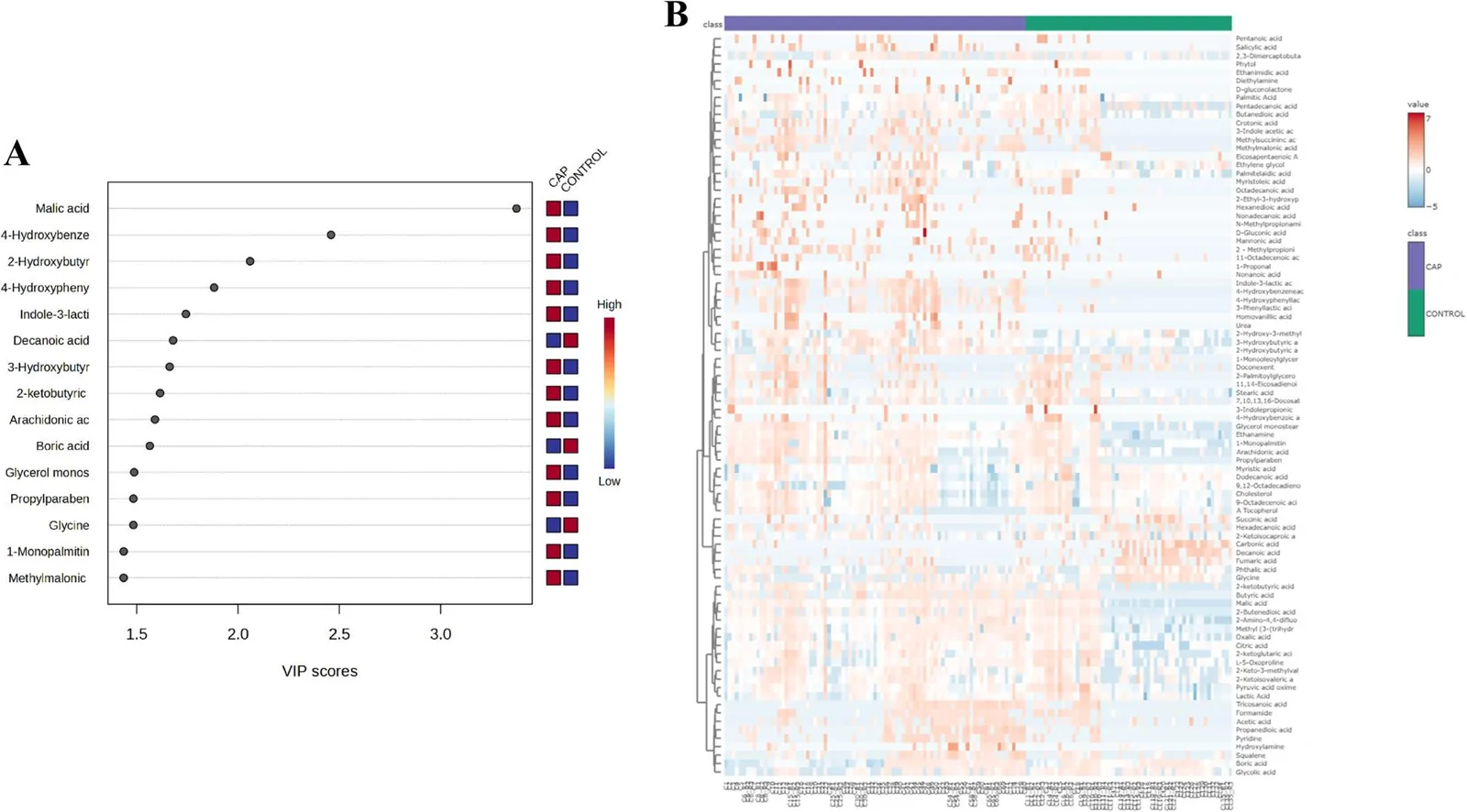
**A** Variable Importance in Projection (VIP) score plot showing key metabolites differentiating CAP and healthy controls. **B** Heatmap of differential metabolites between CAP patients and healthy controls. **A** This plot shows the most significant metabolites contributing to the separation between CAP patients and healthy controls based on OPLS-DA analysis. Higher VIP scores indicate greater importance in distinguishing the two groups, CAP vs control. The colored boxes on the right show whether each metabolite is relatively higher (red) or lower (blue) in CAP compared to controls. **B** This heatmap shows the relative levels of metabolites among CAP patients and healthy controls. Each row represents a metabolite, and each column represents an individual sample. Colours indicate relative abundance (red = higher, blue = lower). Samples are grouped by the categories (CAP and control). The clustering pattern reveals distinct metabolic differences between the two groups

Variable Importance in Projection (VIP) scores were derived from the OPLS-DA models to assess the contribution of individual metabolites to group discrimination. Metabolites with higher VIP scores contributed more strongly to the observed metabolic differences between groups (Fig. 2, Panel A, Table 2). The most potent discriminatory variables identified are malic acid, 4-hydroxybenzeneacetic acid, 2-hydroxybutyric acid, and 4-hydroxyphenyllactic acid. (Fig. 2; Table 2). Heat map–based hierarchical clustering demonstrated a distinct and primary dichotomization between CAP patients and healthy controls, highlighting coordinated alterations in metabolite abundance consistent with a disease-specific metabolic profile. Specifically, a prominent cluster of metabolites, including malic acid, 2-hydroxybutyric acid, and indole-3-acetic acid, showed increased abundance across the CAP cohort. (Fig. 2, Panel B).

**Table 2:** Key dysregulated metabolic pathways and discriminatory metabolites in community-acquired pneumonia (CAP) compared with healthy controls

Pathway	Metabolite	VIP value	Fold change	Log_2_ FC	AUC
Glyoxylate & dicarboxylate metabolism / TCA	Malate or Malic acid	3.374	2.591	1.373	0.790
Aromatic amino acid metabolism	4-Hydroxybenzeneacetic acid (4-HPAA)	2.459	27.97	4.806	0.855
Propanoate metabolism	2-Hydroxybutyrate	2.059	4.779	2.256	0.793
Aromatic amino acid metabolism	4-Hydroxyphenyllactic acid	1.882	20.67	4.369	0.731
Tryptophan metabolism	Indole-3-acetic acid	1.742	10.53	3.39	0.730
Ketone body metabolism	3-Hydroxybutyrate	1.66	7.14	2.83	0.764
Cystine & Methionine metabolism	2-Ketobutyrate	1.61	2.59	1.376	0.768
Arachidonic acid metabolism	Arachidonic acid	1.59	2.05	1.04	0.650
Amino-acid / Propanoate metabolism	Methylmalonate	1.436	3.974	1.991	0.722

*Legend*: Metabolites are ordered according to decreasing Variable Importance in Projection (VIP) scores derived from orthogonal partial least squares–discriminant analysis (OPLS-DA). VIP values reflect the relative contribution of each metabolite to group separation, with higher values indicating greater discriminatory importance. Fold change (FC) represents the ratio of metabolite abundance in community-acquired pneumonia (CAP) patients relative to controls. In contrast, log₂ fold change (log₂FC) indicates the direction and magnitude of change on a logarithmic scale. The area under the receiver operating characteristic (ROC) curve (AUC) reflects the diagnostic performance of individual metabolites in distinguishing CAP from controls, with higher values indicating greater discriminative ability

**Fig. 3:**
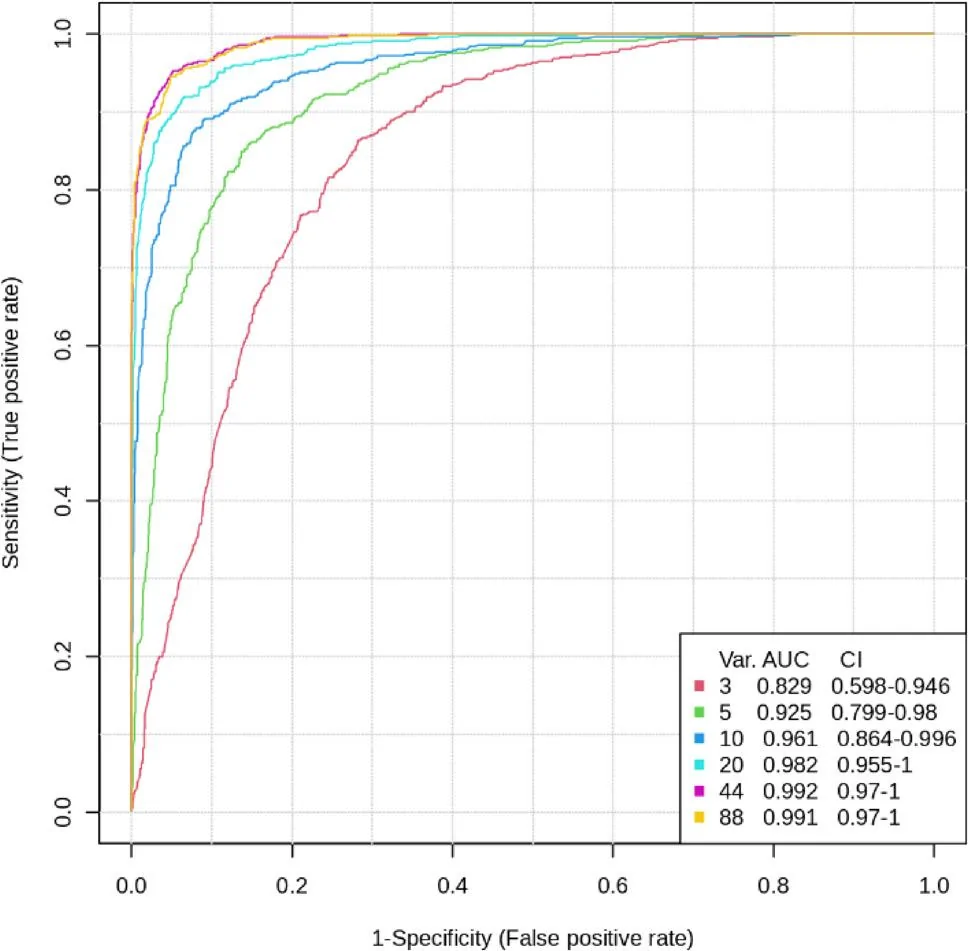
Multivariate ROC curves for metabolite-based discrimination of CAP and healthy controls. Receiver operating characteristic (ROC) curves showing the ability of different metabolites to distinguish CAP patients from healthy controls. Each curve represents a model built using a selected number of variables. The area under the curve (AUC) increases as more metabolites are included, indicating improved diagnostic performance. Sensitivity, 1-specificity, and corresponding AUC values with confidence intervals are depicted

Overall, these findings indicate that a multi-metabolite signature provides strong discriminatory capability between CAP patients and controls, supporting the presence of a distinct disease-associated metabolic profile. (Fig. 3) Multivariate receiver operating characteristic (ROC) analysis was performed to evaluate the ability of a metabolite panel to discriminate CAP patients from healthy controls. (Fig. 3) The ROC curves showed progressive improvement in classification accuracy as the number of metabolites included in the model increased. A panel comprising three metabolites achieved an area under the curve (AUC) of 0.829 (95% CI: 0.598–0.946), indicating moderate discriminatory ability (Fig. 3).

**Fig. 4:**
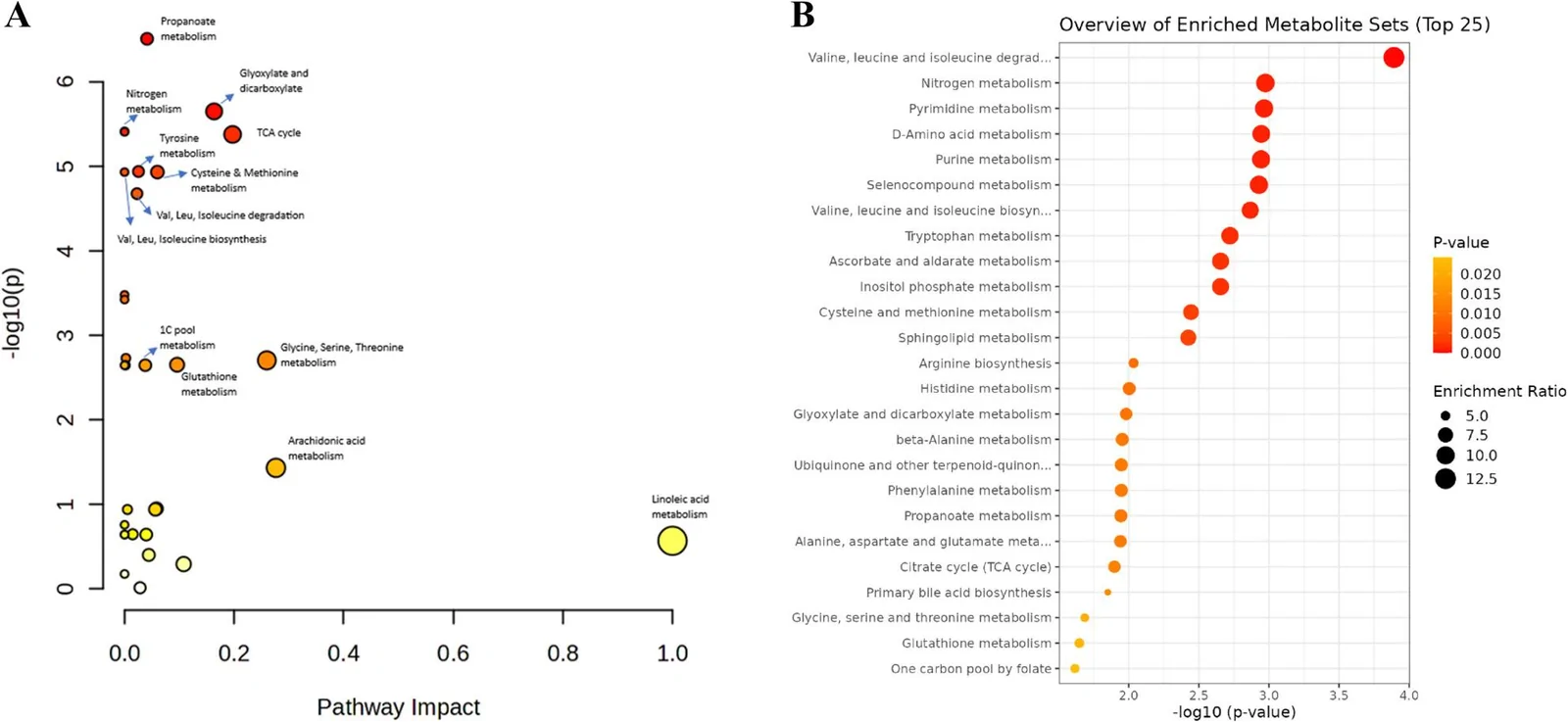
**A** Pathway analysis of differential metabolites in CAP patients as compared to healthy controls. **B** The enriched metabolic pathways identified from differential metabolites between CAP patients and controls. **A **Legend: This bubble plot shows the metabolic pathways altered in CAP compared to controls. Each circle represents a pathway. The X-axis (pathway impact) reflects the relative importance of the pathway, while the Y-axis (-log10 p-value) indicates statistical significance. Larger circles indicate greater pathway impact, and warmer colours (red/orange) indicate more significant changes. Key pathways dysregulated in CAP compared to healthy controls include propanoate. **B** Legend: This plot shows the enriched metabolic pathways identified from differential metabolites between CAP patients and controls. Each dot represents a pathway. The position on the x-axis (-log10 p-value) indicates statistical significance, with values further to the right being more significant. The size of the dot reflects the enrichment ratio (greater size = higher impact), and the colour represents the p-value (red = more significant, yellow = less significant). Key pathways include amino acid, nitrogen, and propionate metabolism

Pathway enrichment analysis identified significant disruption of central metabolic pathways in CAP patients compared with controls (Fig. 4, Panel A). The most prominently affected pathways that were dysregulated in the CAP patients as compared to the healthy controls were propanoate metabolism (raised 2-hydroxybutanoic acid, succinate and 2-oxobutyrate in CAP), glyoxylate and dicarboxylate metabolism pathway (raised malate in CAP), nitrogen metabolism pathway, TCA cycle (raised malate in CAP), tyrosine metabolism (4-hydroxyphenylacetate raised in CAP), cystine and methionine metabolism (increased 2-oxobutanoate in CAP), and valine, leucine and isoleucine degradation.

Although nitrogen metabolism was identified as an enriched pathway, none of the top discriminatory metabolites was core intermediates of nitrogen metabolism. This is likely to reflect increased amino acid turnover and interconnectivity within nitrogen disposal pathways rather than direct alterations in those pathways (Fig. 4 Panel B).

To evaluate smoking as a potential confounder, smokers and non-smokers within the CAP cohort were compared using PCA and OPLS-DA. Of the 70 CAP patients, 19 were smokers; 5 were excluded due to unavailable smoking data. Both analyses demonstrated substantial overlap between groups, and neither plot showed distinct clustering by smoking status. These findings indicated that smoking was unlikely to be a dominant driver of the observed metabolomic profile.

Due to the difference in the age between the CAP patients and healthy controls, an age-adjusted sensitivity analysis was performed on the nine discriminatory metabolites identified in the CAP versus healthy control comparison (Table 3). From the 93 metabolites detected by GC-MS-based untargeted metabolomics, nine were taken forward for age-adjusted multivariable linear regression. Selection was based on Variable Importance in Projection (VIP) scores exceeding 1.0, derived from OPLS-DA for identifying metabolites that contribute meaningfully to group separation. The nine shortlisted metabolites were subsequently examined to determine which retained an independent association with CAP following adjustment for age.Three metabolites retained statistical significance after age adjustment: 2-hydroxybutyrate (β = 0.471, age-adjusted *p* = 0.003, FDR *p* = 0.009), 4-hydroxyphenyl lactic acid (β = 1.074, age-adjusted *p* < 0.001, FDR *p* = 0.003), and 3-hydroxybutyrate (β = 0.821, age-adjusted *p* < 0.001, FDR *p* < 0.001) (Table 3). The remaining six metabolites did not reach significance after FDR correction, suggesting their apparent differences may be partially attributable to age rather than the CAP status.

**Table 3:** Age-adjusted linear regression analysis of selected discriminatory metabolites between CAP patients and healthy controls

Metabolite	Beta	SE	Age-adjusted *p*-value	FDR *p*-value	Interpretation
Malate/Malic acid	0.3622	0.1801	0.047660	0.107234	Nominal only
4-HPAA	0.4000	0.3883	0.307904	0.395877	Not significant
2-Hydroxybutyrate	0.4710	0.1537	0.003	0.009	Significant
4-Hydroxyphenyl lactic acid	1.0740	0.2909	< 0.001	0.003	Significant
Indole-3-acetic acid	0.0958	0.2398	0.692436	0.692436	Not significant
3-Hydroxybutyrate	0.8206	0.1679	< 0.001	< 0.001	Significant
2-Ketobutyrate	0.0695	0.1000	0.489251	0.550407	Not significant
Arachidonic acid	0.2573	0.2093	0.222944	0.334415	Not significant
Methylmalonate	0.1881	0.1442	0.198143	0.334415	Not significant

*Legend*: 4-HPAA:4-Hydroxybenzeneacetic acid. Values represent age-adjusted ordinary least-squares linear regression results for selected discriminatory metabolites identified in the CAP versus healthy control comparison. Beta represents the adjusted difference in log10-transformed metabolite abundance in CAP patients relative to healthy controls; positive beta values indicate higher abundance in CAP. SE: standard error. FDR *p*-values were calculated using the Benjamini–Hochberg method for multiple-testing correction. FDR *p* < 0.05 was considered statistically significant

**Fig. 5:**
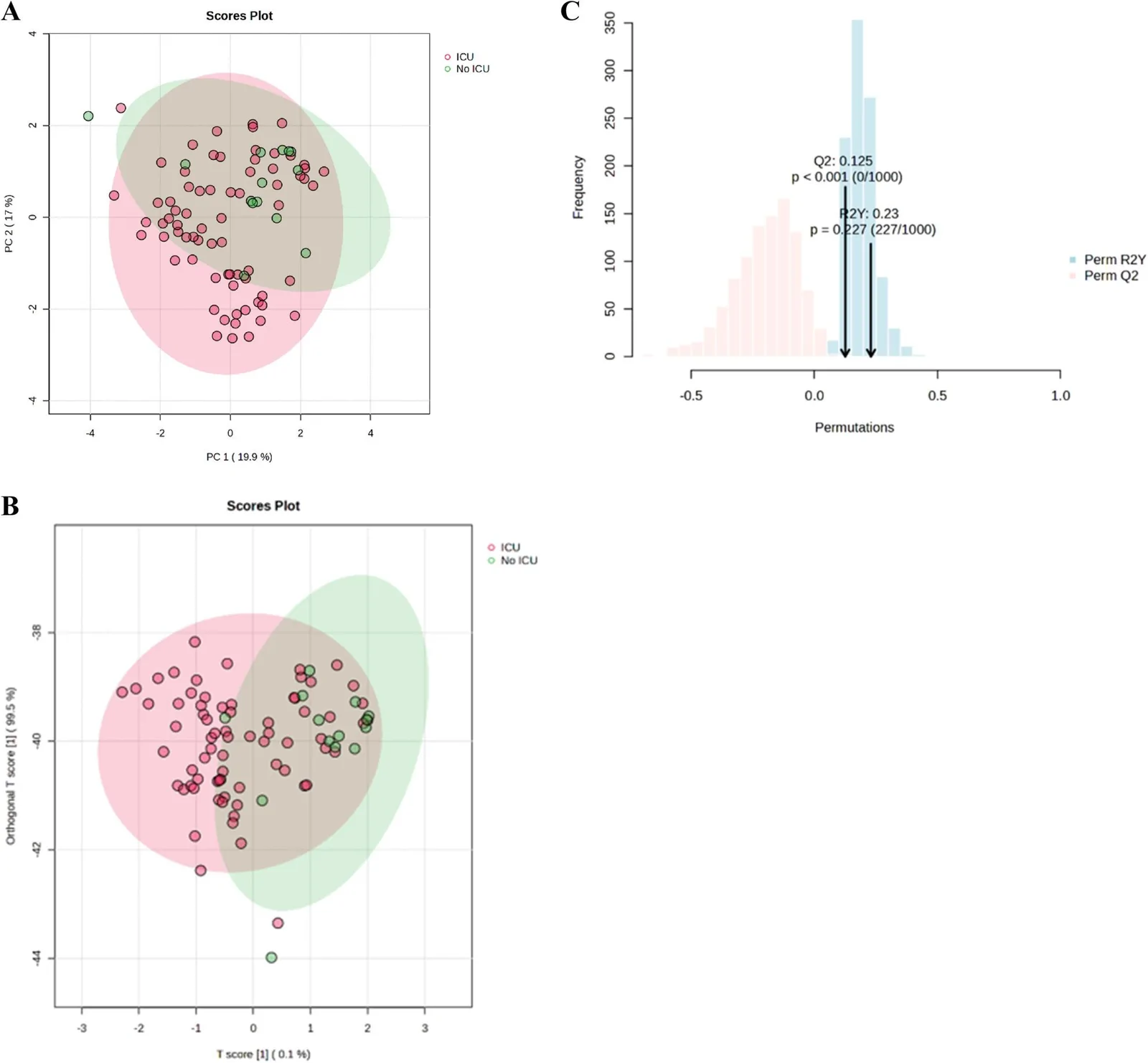
Analysis of metabolic profiles in severe CAP (ICU admission) versus non-severe CAP (no ICU admission). **A** Principal component analysis (PCA) score plot illustrating the global metabolic distribution of severe CAP patients (ICU admitted) and non-severe CAP (non-ICU admitted). **B** Orthogonal partial least squares discriminant analysis (OPLS-DA) score plot demonstrating distinct metabolic separation between severe CAP patients (ICU admitted) and non-severe CAP (non-ICU admitted). **C** Permutation test validating the robustness and reliability of the OPLS-DA model, showing metabolic separation between severe CAP patients (ICU admitted) and non-severe CAP (non-ICU admitted). **A** PCA score plot comparing severe CAP (ICU admission) and non-severeCAP (no-ICU admission) patients.Each point represents an individual CAP patient; red indicates ICU admission and green indicates no ICU admission. PC1 and PC2 explained 19.9% and 17.0% of the variance, respectively. The group ellipses show sample dispersion. Marked overlap between severe CAP (ICU) and non-severe CAP (no-ICU) patients indicates that PCA did not demonstrate clear unsupervised separation based on ICU admission status. **B** OPLS-DA score plot comparing severe CAP (ICU) and non-severe CAP (no-ICU) patients.Orthogonal partial least squares-discriminant analysis (OPLS-DA) score plot showing supervised discrimination of severe CAP (ICU) and non-severe CAP (no-ICU) patients. The plot shows partial separation between ICU and non-ICU patients, but with marked overlap, suggesting limited complete discrimination and heterogeneity of metabolic profiles within both groups. **C** Permutation validation of the OPLS-DA model- comparing severe CAP (ICU) and non-severe CAP (no-ICU) patients.Permutation testing was performed to evaluate the robustness of the OPLS-DA model and assess the risk of overfitting. The histogram shows the distribution of permuted R²Y values in blue and permuted Q² values in pink. The original model showed Q² = 0.125, which was significantly higher than the permuted models (p < 0.001; 0/1000), supporting predictive relevance beyond random class assignment. The R²Y value was 0.23 and was not statistically significant on permutation testing (p = 0.227; 227/1000), indicating modest explanatory performance. Overall, the model showed limited but statistically supported predictive ability, with no strong evidence of overfitting based on Q² permutation testing

We performed the subgroup analysis of the CAP patients to determine the metabolic pathway dysregulations between the severe CAP patients (required ICU admission), and the non-severe CAP patients (no ICU admission). When unsupervised principal component analysis (PCA) was applied, the severe CAP (required ICU admission) and non-severe CAP (non-ICU admitted) there was considerable overlap rather than clear separation. The first two principal components captured 19.9% and 17.0% of total variance respectively, with 36.9% combined. The clustering depicted wide inter-individual variability and the limited power of severe CAP status alone to stratify patients metabolically (Fig. 5, Panel A).

OPLS-DA showed a certain degree of group separation, though the severe CAP and non-severe CAP groups still overlapped substantially, implying that severe CAP patients had only moderate shifts in the circulating metabolome. We applied OPLS-DA to examine class discrimination, and the resulting score plot showed partial separation between groups, but meaningful overlap persisted, and the severe CAP did appear to alter the metabolomic profile as compared to non-severe CAP, though not very prominently (Fig. 5, Panel B).

The OPLS-DA model showed only a very modest model performance, with R²Y of 0.23 and Q² of 0.125. In the permutation test, Q² was statistically significant, *p* < 0.001, whereas R²Y was not significant, *p* = 0.227, indicating that the model showed a non-random prediction, however, does not provide a strong definitive classifier for severe CAP (Fig. 5, Panel C).

**Fig. 6:**
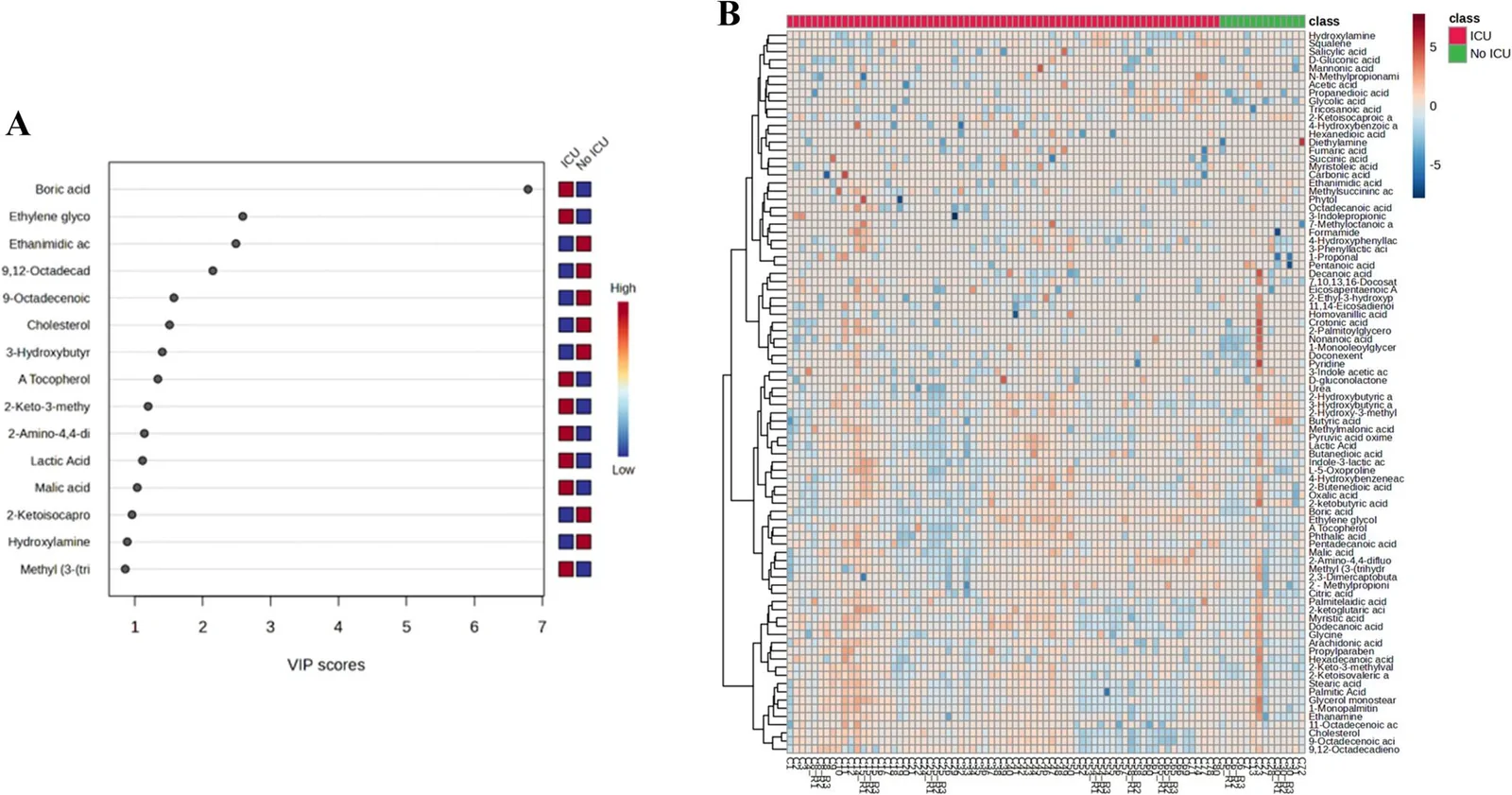
**A** Variable Importance in Projection (VIP) score plot showing key metabolites differentiating severe CAP (ICU admission) and non-severe CAP (no ICU admission). **B** Heatmap of differential metabolites between severe CAP (ICU admission) and non-severe CAP (no ICU admission). **A** Variable importance in projection (VIP) score plot derived from the supervised multivariate model comparing severe CAP patients (ICU admission) with non-severe CAP patients (no ICU admission). Each point represents an individual metabolite, and higher VIP scores indicate greater contribution to group discrimination. Boric acid showed the highest discriminatory contribution, followed by ethylene glycol, ethanimidic acid, 9,12-octadecadienoic acid, 9-octadecenoic acid, cholesterol, 3-hydroxybutyrate, and α-tocopherol. The colour panel on the right indicates relative metabolite abundance in severe CAP (ICU) and non-severe CAP (non-ICU) groups, with red representing higher abundance and blue representing lower abundance. **B** Heat map showing the relative abundance of identified metabolites across CAP patients stratified by severity status. Each column represents an individual patient, and each row represents a metabolite. The top annotation bar indicates clinical class, with red denoting severe CAP (ICU admission) patients and green denoting non-severe CAP (no ICU admission patients). Metabolite abundance is shown as scaled intensity, with red indicating higher relative abundance and blue indicating lower relative abundance. Hierarchical clustering of metabolites is displayed on the left, grouping metabolites with similar expression patterns. The heat map demonstrates heterogeneous metabolomic profiles across severe CAP (ICU admission) and non-severe CAP (no ICU admission) patients, with overlapping abundance patterns and no complete class-wise separation, consistent with the partial discrimination observed in supervised score plots

VIP analysis was used to determine the metabolites most responsible for separating the severe CAP and non-severe CAP groups. Among them, boric acid (VIP score 6.78), ethylene glycol (VIP score 2.58), ethanimidic acid (VIP score 2.48), 9,12-octadecadenoic acid (VIP score 2.14), 9-octadecenoic acid (VIP score 1.57), cholesterol (VIP score 1.51) and alpha-tocopherol were the key VIP-ranked discriminatory metabolites (Fig. 6, Panel A) in the severe CAP group. The FC values of boric acid, ethylene glycol and alpha tocopherol were 3.28, 2.23 and 2.00 respectively, showing higher abundance in severe CAP patients. These compounds implicate oxidative stress, ketone body turnover, and energy balance, processes consistent with the greater physiological demands seen in severe CAP patients compared to the non-severe CAP patients.

Hierarchical clustering showed that metabolite clusters had differential abundance between severe CAP and non-severe CAP groups, but no clear discrimination emerged from the heat map. The broad overlap in expression profiles across both groups echoed the PCA, and OPLS-DA findings, implying how metabolically diverse CAP severity can be. (Fig. 6, Panel B).

**Fig. 7:**
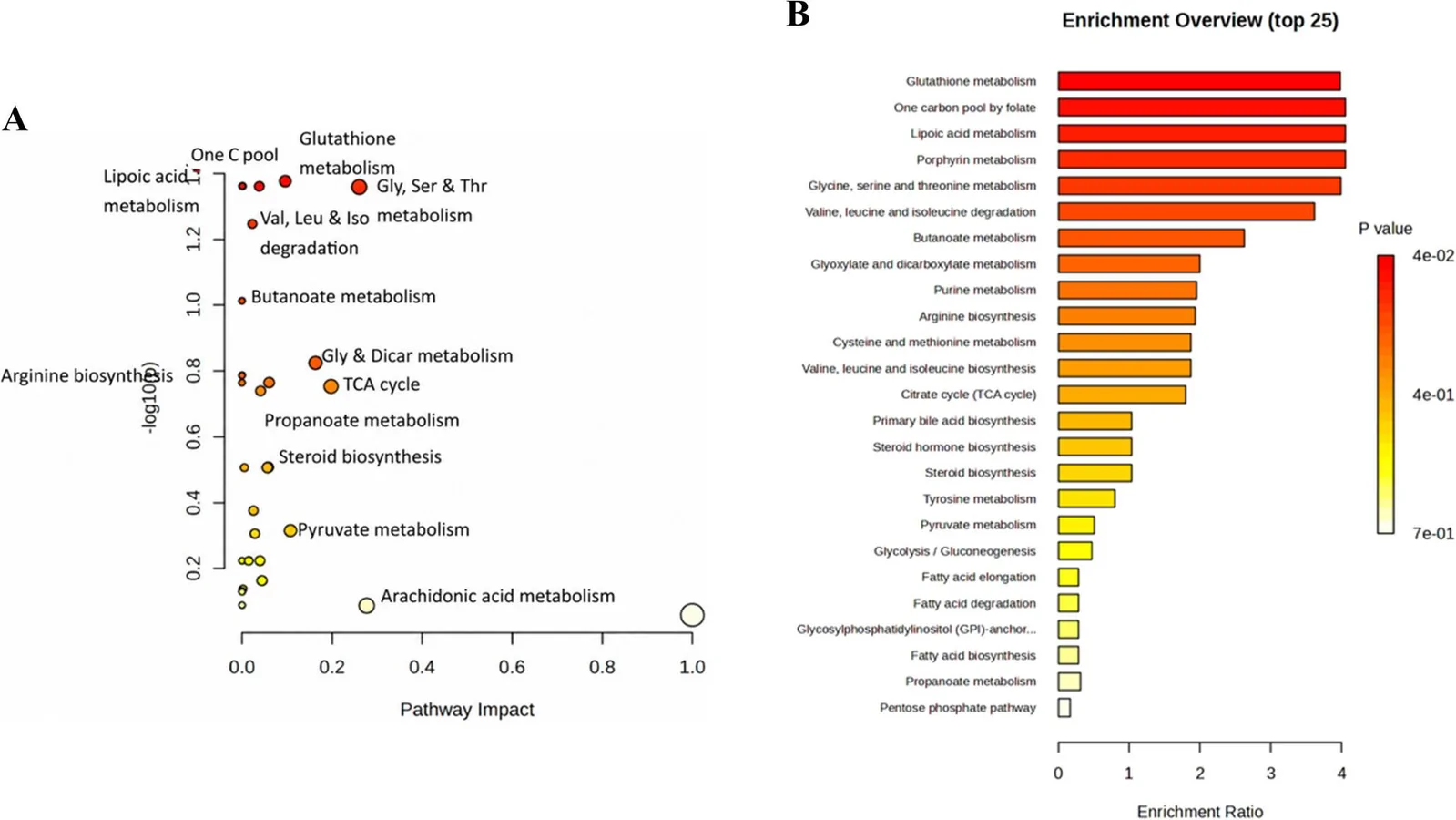
**A** Pathway analysis of differential metabolites in severe CAP patients (ICU admission) as compared to non-severe CAP patients (no-ICU admission). **B** The enriched metabolic pathways identified from differential metabolites between severe CAP patients (ICU admission) as compared to non-severe CAP patients (no-ICU admission). **A **Pathway enrichment and topology analysis comparing severe community-acquired pneumonia (CAP) requiring intensive care unit (ICU) admission with CAP not requiring ICU admission.The most significantly enriched pathways included glutathione metabolism, glycine-serine-threonine metabolism, lipoic acid metabolism, valine-leucine-isoleucine degradation, butanoate metabolism, glyoxylate and dicarboxylate metabolism, the tricarboxylic acid (TCA) cycle, propanoate metabolism, pyruvate metabolism, steroid biosynthesis, and arachidonic acid metabolism. **B **The enrichment overview shows the top 25 metabolic pathways enriched in severe CAP compared with non-severe CAP. The X-axis represents the enrichment ratio, and bar colour indicates pathway-level significance, with red denoting lower p-values. The most enriched pathways included glutathione metabolism, one-carbon metabolism, lipoic acid metabolism, glycine/serine/threonine metabolism, branched-chain amino-acid degradation, glyoxylate and dicarboxylate metabolism, and the TCA cycle

Pathway enrichment analysis pointed to a range of disrupted metabolic processes in severe CAP. These included glutathione metabolism, glycine-serine-threonine metabolism, branched-chain amino acid degradation, butanoate metabolism, glyoxylate and dicarboxylate metabolism, the TCA cycle, propanoate and pyruvate metabolism, steroid biosynthesis, and arachidonic acid metabolism. (Fig. 7, Panels A, B). Collectively, these shifts implicate impaired redox balance, altered energy production, amino acid breakdown, and pro-inflammatory lipid signalling as features tied to severe CAP patients.

**Fig. 8:**
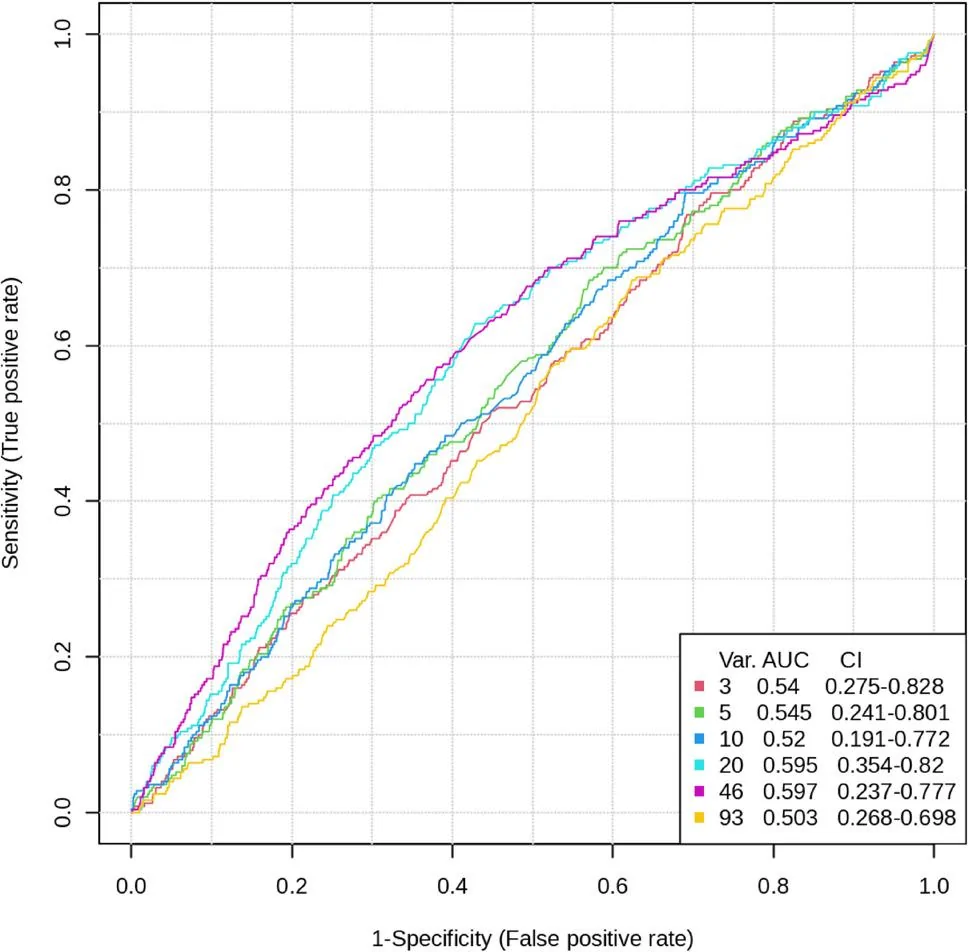
Multivariate ROC analysis for severe CAP patients (ICU admission) classification in CAP. Legend: Multivariate receiver operating characteristic (ROC) curves showing the performance of metabolite panels of different sizes in discriminating against severe CAP patients requiring ICU admission from non-severe CAP (not requiring ICU admission). The x-axis represents 1-specificity, false positive rate, and the y-axis represents sensitivity, true positive rate. Models using 3, 5, 10, 20, 46, and 93 variables showed AUC values of 0.540, 0.545, 0.520, 0.595, 0.597, and 0.503, respectively. Overall, the AUC values were close to 0.5, indicating limited discriminatory ability of the metabolite panels for predicting ICU admission status in CAP patients

However as compared to the distinct CAP-versus- healthy control discrimination, the severe CAP versus non-severe CAP based ROC analysis showed modest discriminatory performance, with AUC values of 0.540, 95%CI [0.275–0.828] for a 3-metabolite model, and 0.595, 95%CI [0.354–0.820] for a 20-metabolite model (Fig. 8). These findings indicate that although the severe CAP subgroup did not generate a very distinctive predictive classifier, it showed biologically meaningful enrichment of pathways related to redox imbalance, mitochondrial dysfunction, antioxidant response, one-carbon metabolism and amino-acid catabolism.

## Discussion

CAP patients have a risk of hypoxemia severity progression and require early identification of the pathophysiological process to prevent it, with an array of investigative techniques [18]. In this study, we identified early and distinct metabolomic alterations in CAP patients compared with healthy controls, highlighting clinically relevant pathway disruptions. Key discriminatory metabolites reflected dysregulation of propanoate metabolism, glyoxylate–dicarboxylate metabolism, the TCA cycle, and amino acid pathways, indicating early oxidative stress, mitochondrial dysfunction, and altered energy metabolism.

Though extensive literature is available on the utility of metabolomics in patients of CAP through studies done by Banoei MM et al., the aims and objectives of the previous studies were different to that of ours [12, 13]. Banoei MM et al. in 2017 had examined if serum metabolomics was having diagnostic and prognostic utility in H1N1 pneumonia, and found that early serum metabolomic profiling could differentiate H1N1 influenza pneumonia from bacterial CAP and ventilated ICU controls, and could also predict 90-day mortality in H1N1 pneumonia [12]. The authors found that H1N1 pneumonia is accompanied by metabolomic changes in the concentration of common metabolites such as amino acids and ketone bodies [12]. In 2020, Banoei MM et al. had examined if the quantitative lipid profiling can predict the 90-day mortality and in-hospital mortality among patients with bacterial CAP. The study had concluded that decreased lysophosphatidylcholines and phosphatidylcholines with increased acylcarnitines, was associated with 90-day mortality, in-hospital mortality, ICU admission and PSI-based CAP severity [13].

In our study, we wanted to determine the early metabolic alterations in all-cause CAP patients with an untargeted metabolomic analysis approach rather than a specific lipidomic prognostic signature, to identify broader pathway-level disruptions energy metabolism, amino-acid metabolism, redox imbalance, lipid-inflammatory signalling and gut–host metabolic axes. The novelty in our study is in the fact that we could identify early untargeted metabolic disruptions that begin early in hospitalized CAP patients. We also did an exploratory analysis of the metabolic alterations in the severe CAP subgroup and could find that the progression from CAP to severe CAP can involve metabolomic alterations beyond lipid profiles, and may include oxidative stress, compensatory antioxidant response, hypoxia-driven mitochondrial dysfunction, gut-barrier or microbiota-related metabolic perturbation and hypercatabolic amino-acid utilisation. Thus, the novelty of our study lies in extending previous metabolomic observations from viral pneumonia discrimination and lipid-based bacterial CAP prognosis to an untargeted, pathway-level metabolic phenotype of severe CAP, providing an exploratory additional insight into early biochemical changes associated with clinical deterioration.

We found that the propanoate metabolism is the most dysregulated in CAP patients. The rise in 2-hydroxybutyrate in the propionate pathway in CAP compared with healthy controls indicates early oxidative stress, redox imbalance, and mitochondrial dysfunction during physiological stress [19–21]. The dysregulation of the glyoxylate dicarboxylate (TCA) cycle, with accumulation of malate, was another important finding in CAP patients compared with healthy controls. This may be due to mitochondrial dysfunction and cellular hypoxia resulting from increased utilisation of adenosine triphosphate (ATP) during acute illness, leading to the accumulation of intermediates such as malate [22, 23]. The metabolic pathways reflecting early mitochondrial dysfunction, as we found in CAP patients, were also reported by Langley et al. in sepsis patients, who concluded that hypoxia is a clinical signal of mitochondrial dysfunction [24]. Decreased ATP production and dysregulated mitochondrial complex expression during Streptococcus pneumoniae-induced oxidative stress have also been reported in previous literature [25].

We also found elevated 3-hydroxybutyrate (BHB) and arachidonic acid (AA) in early CAP, which may reflect metabolic stress from hypoperfusion, inflammation, and gut dysbiosis [26–29]. Increased AA-derived eicosanoids lead to inflammatory acute lung injury, as shown by Lu X et al. [26] Enhanced phospholipase A2 activity releases AA from membrane phospholipids, which is subsequently metabolised into pro-inflammatory eicosanoids such as prostaglandins and leukotrienes [26]. These lipid mediators amplify neutrophilic inflammation and disrupt alveolar epithelial function [26]. This may explain how the alterations in AA metabolism may lead to early alveolar dysfunction in CAP patients.

The concurrent dysregulation of ketone body metabolism and arachidonic acid signalling at an early stage of CAP indicates early infection, inflammation and lung injury [24, 27, 30, 31]. Hypoxemia and cytokine-mediated insulin resistance concurrently impair glucose oxidation, inducing hepatic upregulation of 3-hydroxy-3-methylglutaryl-CoA (HMG-CoA) synthase to promote ketogenesis and raise 3-hydroxybutyrate levels [30].

Alterations in 4-hydroxyphenylacetic acid (4-HPAA) via dysregulation of aromatic amino acid metabolism in CAP reflect altered gut–liver–host metabolic interactions during acute infection and systemic stress, as seen in septic shock [31, 32]. This could explain why we also observed a rise in 4-HPAA levels in CAP patients. Dysbiosis and increased gut permeability in sepsis can elevate circulating 4-HPAA levels [31, 32]. In CAP, systemic inflammation and increased gut permeability promote enhanced aromatic amino acid catabolism and translocation of microbial metabolites into circulation [31, 32].

Emerging evidence indicates that gut microbiota dysbiosis occurs in CAP and may contribute to systemic inflammation [33, 34]. Patients with CAP have been shown to exhibit alterations in gut microbiota, including the loss of short-chain fatty acid–producing commensals and the enrichment of pro-inflammatory bacteria, compared with healthy controls [34]. This dysbiosis is associated with impaired intestinal barrier integrity, facilitating translocation of bacterial products such as lipopolysaccharide (LPS) into the circulation, which in turn activates systemic inflammatory pathways and elevates cytokines such as tumour necrosis factor alpha (TNF-α) and interleukin-6 (IL-6) [35, 36]. Such systemic immune activation can exacerbate pulmonary inflammation and compromise alveolar barrier function [35, 36]. Recognition of gut dysbiosis in CAP signifies the gut–lung axis in modulating disease severity and opens avenues for microbiota-targeted preventive and therapeutic strategies [34–36].

The increased degradation of essential branched-chain amino acids (BCAAs) valine, isoleucine, and leucine reflects a hypercatabolic state with accelerated muscle proteolysis and diversion of BCAAs toward energy production [37, 38]. Dynamic alterations in amino acid levels are seen in sepsis [38]. This BCAA degradation pattern has been linked to greater illness severity and poorer outcomes in sepsis, supporting its interpretation as an early signature of metabolic stress in CAP [37]. Importantly, these metabolic findings align with the concept that patients at higher risk of malnutrition experience worse ICU outcomes, signifying that metabolic catabolism and nutritional vulnerability frequently coexist during severe infection [39]. Thus, combining evidence of amino-acid catabolism helps in the identification of malnutrition risk, and the timely initiation of nutrition therapy in patients who are biologically prone to deterioration is essential [39, 40]. Early enteral nutrition with adequate protein delivery is essential to mitigate cumulative protein deficits, preserve lean body mass, and reduce complications in high-risk infected patients [40].

We also found dysregulation of tryptophan metabolism and increased levels of indole-3-acetic acid (IAA) in CAP patients compared with healthy controls. Our findings may be explained by recent population-based cohort studies, which show that higher baseline IAA predicts pneumonia hospitalisation risk [41]. In severe CAP patients, elevated IAA increases pulmonary damage and even mortality independent of confounders [41]. Mouse pneumonia models also showed IAA worsens lung damage via neutrophil ROS [41].

Our study on CAP patients was conducted in a manner similar to that of Xu J et al., who aimed to determine “functional metabolites” in 42 ARDS patients compared to 28 healthy controls and found that phenylalanine-tyrosine metabolism was altered in non-survivors [42]. We also aimed to determine the early metabolic dysregulation in CAP patients.

Elevated metabolite levels that indicate increased amino-acid catabolism, upregulation of the trans-sulfuration pathway during inflammation, and accumulation of 4-hydroxyphenyllactic acid are all indicators of tissue hypoxia, mitochondrial dysfunction, and gut dysbiosis, and this is probably quite profound even in the early stages of CAP, as we found [43–45]. The summary of the clinical significance of the findings of this study and the possible reasons why the metabolites were raised in CAP patients are depicted in Additional File 1. Our findings indicate the potential future value of metabolomics for early risk stratification and possibly as a dynamic marker of future therapeutic response.

One important highlight of our study is the early dysregulation of metabolic pathways for aromatic amino acids and branched-chain amino acids (BCAAs), involving the gut-lung axis. Recent literature supports our findings in CAP. Recent literature has shown enrichment of Gemmiger and Enterocloster in CAP, reflecting dysbiotic metabolic shift toward amino acid fermentation, accompanied by BCAA metabolism [46]. This altered microbial metabolism is characterised by accumulation of phenolic and aromatic acids, increased BCAA, and depletion of protective tryptophan-derived indole metabolites [47].

In severe CAP patients compared with non-severe CAP patients, we found that boric acid, ethylene glycol, and alpha-tocopherol were upregulated. Severe CAP is associated with systemic inflammation–induced gut barrier dysfunction, leading to increased intestinal permeability and translocation of bowel lumen metabolites into the circulation [48]. Raised levels of boric acid that are derived from dietary and microbiota-associated sources may be due to gut leak and dysregulated host–microbial metabolic interactions in severe CAP [49]. Elevated ethylene glycol–like metabolites likely reflect hypoxia-driven mitochondrial dysfunction and enhanced anaerobic metabolism [50]. Increased α-tocopherol represents a compensatory antioxidant response to heightened oxidative stress and lipid peroxidation, suggesting greater cellular injury in severe disease [51, 52]. Additionally, disrupted glutathione and lipoic acid metabolism indicate impaired redox homeostasis and mitochondrial dysfunction [53, 54] Dysregulation of glycine, serine, and threonine metabolism further reflects a hypercatabolic state with increased amino acid utilisation for antioxidant defence and cellular repair processes [55]. We performed an exploratory study in a novel area of untargeted metabolomics, analysing 93 metabolites in 70 CAP patients to detect early changes compared with healthy controls. We could highlight that oxidative stress, amino acid breakdown, alveolar damage, and gut-metabolic dysbiosis start early in CAP.

However, there were limitations. Although we obtained the serum samples within 24 h of hospitalization, the drug therapies administered could have altered patients’ metabolic pathways, as we could not ensure that all serum samples were obtained strictly before any drug was administered as a part of the immediate acute care post hospitalization. We did not perform sequential metabolomics to detect trends in response to therapies.

The use of a single early time point should be interpreted in relation to the primary objective of the study. This study aimed to identify early metabolomic alterations in CAP at hospital presentation rather than to describe longitudinal changes during illness. Therefore, serum sampling within 24 h of admission provides a clinically relevant early metabolic picture, reflecting the host metabolic state near the time of diagnosis and initial risk stratification. Similar early-sampling approaches have been used in previous pneumonia metabolomics studies, where admission or first-day samples were analysed to identify diagnostic or prognostic metabolic signatures [13]. However, the single-time-point assessment cannot define whether these metabolic abnormalities persist, resolve or worsen over time with the progression of disease in the CAP patients.

The bacteria/virus causing CAP could have altered metabolism, and we could not account for that. The specific nutritional status and enteral diet of the patients were not taken into consideration, as they could have affected the metabolic pathways. Because we performed untargeted metabolomics, we could only compare levels relative to healthy controls, not quantify serum levels in absolute concentration. The sample was relatively small, comprising 70 CAP patients, and generalizability may be limited. The relatively small number of non-ICU CAP patients may limit the generalizability of severity-based comparisons. Another limitation of this study is that the ROC analyses performed reflect apparent within-sample discriminatory ability rather than validated diagnostic or prognostic model performance. Given the modest sample size splitting the cohort into separate training and testing datasets was not feasible, as this would have yielded small, statistically unstable subgroups and increased the risk of overfitting, a concern that was particularly pronounced for the severe versus non-severe CAP subgroup analysis. Accordingly, the reported AUC values should be interpreted with caution and the metabolomic signatures identified here require prospective validation in larger, independent cohorts before any clinical utility can be established.

## Conclusion

We identified early, coordinated derangements across multiple metabolic cycles in CAP, including dysregulated propanoate, glyoxylate–dicarboxylate, TCA energy, tyrosine, cystine-methionine, and BCAA cycles. Collectively, these changes indicate early metabolic stress characterised by hypercatabolic, redox imbalance, mitochondrial dysfunction, amino-acid breakdown, and inflammatory lipid signalling with alveolar epithelial injury in CAP.

### Clinical significance

CAP is associated with early, coordinated metabolomic disruptions involving energy, amino-acid, redox, lipid, inflammatory, and gut–host pathways. These findings support the clinical utility of metabolomic profiles as an adjunctive tool for early risk stratification in patients with community-acquired pneumonia.

## Supplementary Information

Supplementary Material 1.

## Data Availability

The datasets used and/or analysed during the current study are available from the corresponding author on reasonable request.

## Abbreviations

AA: Arachidonic acid
AKI: Acute kidney injury
ALT: Alanine aminotransferase
ARDS: Acute respiratory distress syndrome
AST: Aspartate aminotransferase
ATP: Adenosine triphosphate
AUC: Area under the curve
BCAA: Branched-chain amino acids
BHB: Beta-hydroxybutyrate
CAP: Community-acquired pneumonia
CCI: Charlson comorbidity index
CI: Confidence interval
CRP: C-reactive protein
CURB-65: Confusion, urea, respiratory rate, blood pressure and age ≥ 65 years
CXR: Chest radiograph
EF: Ejection fraction
FC: Fold change
GC: Gas chromatography
GC-MS: Gas chromatography–mass spectrometry
GCS: Glasgow coma scale
Hb: Haemoglobin
HbA1C: Glycated haemoglobin
HMG-CoA: 3-hydroxy-3-methylglutaryl-coenzyme A
IAA: Indole-3-acetic acid
ICU: Intensive care unit
IEC: Institutional ethics committee
IL-6: Interleukin-6
IMV: Invasive mechanical ventilation
IQR: Interquartile range
LC-MS: Liquid chromatography–mass spectrometry
LPS: Lipopolysaccharide
MS: Mass spectrometry
NIST: National institute of standards and technology
NIV: Non-invasive ventilation
OPLS-DA: Orthogonal partial least squares–discriminant analysis
PaO₂/FiO₂: Partial pressure of arterial oxygen to fraction of inspired oxygen ratio
PCA: Principal component analysis
PSI: Pneumonia severity index
ROC: Receiver operating characteristic
ROS: Reactive oxygen species
RR: Respiratory rate
SBP: Systolic blood pressure
SD: Standard deviation
SMART-COP: Systolic blood pressure, multilobar infiltrates, albumin, respiratory rate, tachycardia, confusion, oxygenation, and arterial pH
SOFA: Sequential organ failure assessment
TCA: Tricarboxylic acid
TNF-α: Tumour necrosis factor alpha
VIP: Variable importance in projection
WBC: White blood cell
