# Supplementary material for: Metabolomic signatures of early pathway disruptions and clinical outcomes in community-acquired pneumonia: a prospective case-control study
Source: BMC Pulm Med. 2026 Jul 23;26:440. doi: 10.1186/s12890-026-04503-9 (PMC13625439; doi:10.1186/s12890-026-04503-9)
Supplement: Supplementary file 1 [file 12890_2026_4503_MOESM1_ESM.docx]

Supplementary Table 1: Table depicting the dysregulated metabolic pathway in CAP patients as compared to the healthy controls, the possible rationale and the clinical relevance

| Pathway | Metabolite | Direction in CAP | Biochemical Cycle / Source | Pathophysiological Rationale | Clinical Implications (References) |
| --- | --- | --- | --- | --- | --- |
| Propanoate metabolism | 2-Hydroxybutyrate | Increased | Threonine & methionine metabolism; glutathione pathway | Reflects increased cysteine demand for glutathione during oxidative stress and mitochondrial dysfunction.  In CAP, oxidative stress and systemic inflammation increase glutathione demand, leading to elevated 2-HB as a by-product of enhanced amino-acid catabolism and redox adaptation | Marker of redox imbalance and disease severity.  (16-18, 21,22) |
| Aromatic amino acid metabolism | 4-Hydroxybenzeneacetic acid or 4-hydroxy phenyl acetic acid (4-HPAA) | Increased | Tyrosine/phenylalanine metabolism and gut microbiota | 4-HPAA is a microbial-derived phenolic metabolite arising from aromatic amino acid metabolism, reflecting gut–liver–host metabolic interactions that are perturbed in infection and systemic stress. Dysbiosis and increased gut permeability in sepsis can elevate circulating 4-HPAA.  4-HPAA is a major colonic microbiota-derived metabolite, which can be absorbed into blood circulation | Elevated levels reflect gut permeability, dysbiosis, and systemic inflammatory stress  (29,30) |
| Cystine & methionine metabolism | 2-ketobutyrate | Increased | By-product of methionine & threonine degradation, activation of the trans-sulfuration pathway in response to oxidative stress. | Indicates increased amino-acid catabolism, tissue hypoxia, and oxidative stress; generated when the trans-sulfuration pathway is upregulated during inflammation | Marker of systemic catabolic stress and severity (40) |
| Glyoxylate & dicarboxylate metabolism / TCA | Malate or Malic acid | Increased | TCA cycle; malate–aspartate shuttle | Accumulates due to mitochondrial dysfunction and altered NADH/NAD⁺ balance during hypoxia, altered anaplerotic metabolism. | Marker of mitochondrial failure, cellular hypoxia (19, 20,21) |
| Aromatic amino acid  (phenylalanine and tyrosine metabolism) | 4-hydroxy phenyl lactic acid  (4-HPLA) | Increased | 4-Hydroxyphenyllactic acid (4-HPLA) is a downstream product of tyrosine metabolism | 4-HPLA rises in acute infection, pneumonia, and sepsis, where pathobionts overproduce tyrosine-derived metabolites while failing to bio transform them. | Elevated 4-HPLA has been consistently associated with **disease severity, organ dysfunction, and mortality in sepsis**, supporting its role as a **severity-linked metabolic biomarker** relevant to CAP patients at risk of deterioration.  (40-42) |
| Amino-acid / Propanoate metabolism | Methylmalonate | Increased | Product of propionyl-CoA metabolism; vitamin B12-dependent | Increased due to enhanced BCAA and odd-chain fatty-acid breakdown, mitochondrial dysfunction, during inflammation | Marker of metabolic oxidative stress and mitochondrial dysfunction (44) |
| Ketone body metabolism | 3-Hydroxybutyrate  (β-hydroxybutyrate) | Increased | Ketone body from hepatic fatty-acid oxidation | Reflects increased lipolysis, insulin resistance, and stress metabolism during infection | Contributes to alveolar dysfunction and hypoxemia; severity marker (23-26) |
| Arachidonic acid metabolism | Arachidonic acid | Increased | Membrane phospholipid metabolism → eicosanoid pathway | Arachidonic acid is released from cell membrane phospholipids by phospholipase A2 during infection and inflammation. In CAP and sepsis, immune activation and cellular injury lead to increased AA availability as a substrate for inflammatory mediator synthesis, and leads to alveolar epithelial injury | Elevated AA reflects active inflammatory lung injury and has been associated with disease severity, progression to ARDS, and adverse outcomes in pneumonia and sepsis, supporting its role as a clinically relevant inflammatory biomarker. (23-28) |
| Tryptophan metabolism | Indole-3-acetic acid (IAA) | Increased | Tryptophan metabolism | Dysregulated tryptophan metabolism during infection states, enhanced indole production by gut microbes, and increased intestinal permeability | IAA increased pulmonary damage in pre-clinical models. (38) |
